# Supplementary material for: Association of neighborhood disadvantage with cognitive function and cortical disorganization in an unimpaired cohort
Source: ArXiv. 2024 Jun 19:arXiv:2406.13822v1. Preprint. [Version 1] (PMC11213155)
Supplement: Supplement 1 [file NIHPP2406.13822v1-supplement-1.pdf]

Supplementary Materials:

Cortical thickness	Intercept	ADI	Age	Gender	Education
i. Entorhinal_R (Ent.R)	3.81 (0.14)	-0.1 (0.04)	-0.005 (0.001)	0.03 (0.03)	0.001 (0.006)
	p<0.0001	p<0.01	p<0.005	p=0.243	p=0.832
ii. Fusiform_L (Ffm.L)	2.66 (0.05)	-0.007 (0.01)	-0.002 (0.0005)	-0.003 (0.01)	0.0007 (0.002)
	p<0.0001	p<0.584	p<0.0001	p=0.733	p=0.721
iii. Lateralorbitofrontal_L (lOFr.L)	2.81 (0.05)	-0.001(0.01)	-0.003 (0.0006)	-0.01(0.01)	0.004 (0.002)
	p<0.0001	p=0.905	p<0.0001	p=0.147	p<0.05

Table-S1: Results of regression models for association between neighborhood disadvantage (ADI) and average cortical thickness at (i) significance of $p<0.01$ and for regions (ii,iii) which showed significance ($p<0.01$) on local MSN features , with age, gender (coded as male-1, female-0), education(years) as covariates. Results represented as β-coefficients (Standard Error).

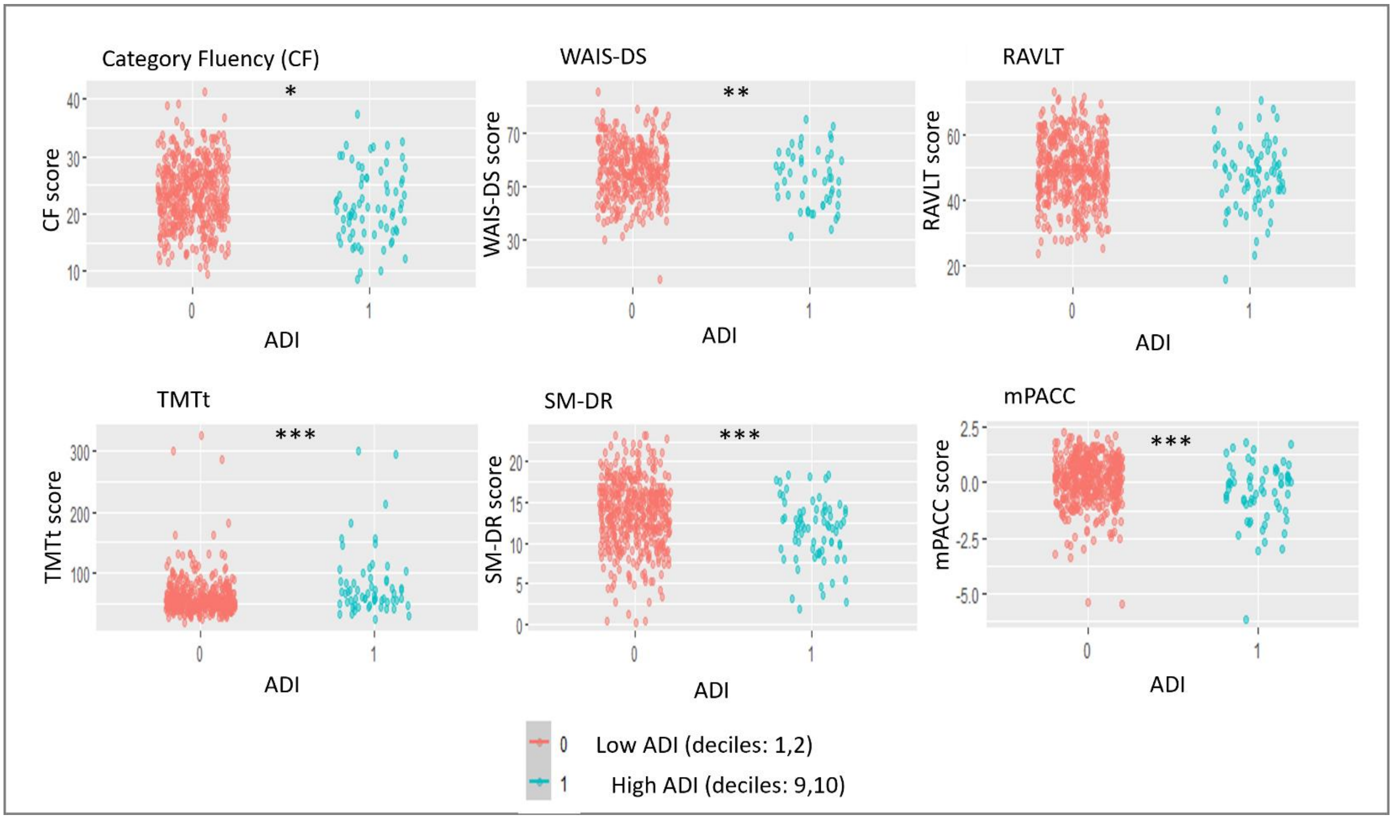

Figure-S1: Plots depicting distribution of cognitive scores across for low (decile-1,2) and high (decile-9,10) ADI populations. Significant associations between ADI and cognitive scores obtained using regression models: Cognitive score (y) ~ ADI (x) + Age+Gender+Education+Practice effect (covariates) are indicated $*p<0.05$, $**p<0.01$, $***p<0.001$ in the above plot.

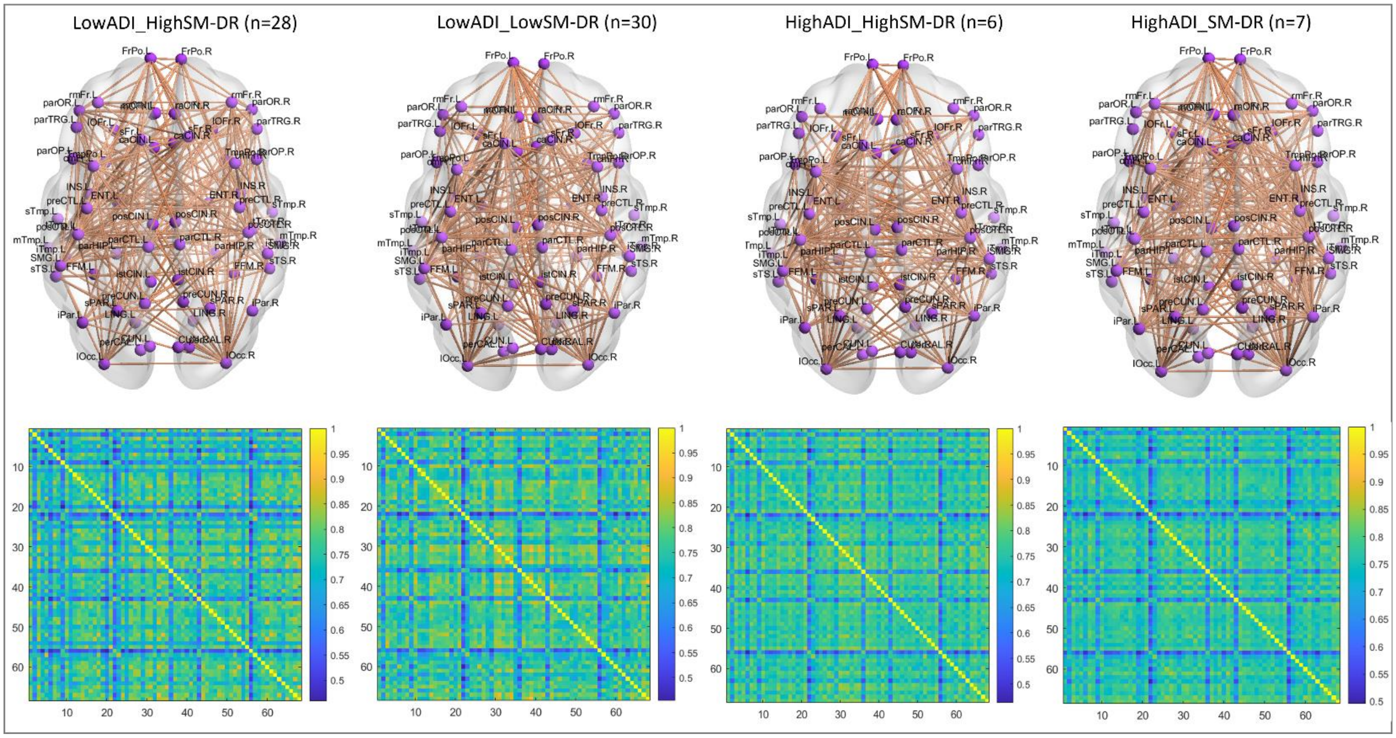

Figure-S2: Visualization of MSN graphs stratified by ADI (low-deciles0,1; high-deciles 9,10) and cognitive performance on SM-DR tests, with top row indicating top 10% of edges with high value, while bottom row indicates the fully dense graph representing all edge values.
